# Necrotizing streptococcal myositis of the upper extremity: a case report

**DOI:** 10.1186/s13104-017-2743-1

**Published:** 2017-08-15

**Authors:** Johannes C. Reichert, Götz Habild, Paul Simon, Ulrich Nöth, Jan B. Krümpelmann

**Affiliations:** 1 Department of Orthopaedic and Trauma Surgery, Ev. Waldkrankenhaus Spandau, Stadtrandstr. 555, 13589 Berlin, Germany; 2 Department of Orthopaedic Surgery, König-Ludwig-Haus, Center for Musculoskeletal Research, Julius-Maximilians-University, Brettreichstraße 11, 97074 Würzburg, Germany

**Keywords:** β-Hemolytic streptococcus, Infection, Necrotizing myositis, Upper extremity, Plastic surgery

## Abstract

**Background:**

Necrotizing myositis is a rare but life-threatening soft-tissue infection characterized by rapidly spreading inflammation and subsequent necrosis of the affected tissue. The myositis is often caused by toxin-producing, virulent bacteria such as group A β-hemolytic streptococcus and associated with severe systemic toxicity. It is rapidly fatal unless diagnosed promptly and treated aggressively. However, necrotizing myositis is often initially misdiagnosed as a more benign soft-tissue infection as such fulminant, invasive muscle infections are rare with no more than 30 cases reported over the last century.

**Case presentation:**

We illustrate the case of a 74-year-old male Caucasian initially presenting with a progressing swelling and gradually oncoming pain of the upper right extremity. Rapidly, livid discolorations of the skin, blisters, hypoesthesia and severe pain resistant to analgesics treatment developed accompanied by disruption of the arterial blood flow. Due to a manifest compartment syndrome the patient was admitted to theater for fasciotomy of the arm. After multiple revision surgeries wound closure was achieved using a pedicled, fasciocutaneous parascapular flap and a free, ipsilateral anterolateral thigh flap. Microbiological analysis revealed group A β-hemolytic streptococcus, histology a bacterial interstitial myositis with necrotic muscular fibers.

**Conclusions:**

A high degree of clinical suspicion is necessary to avert potentially disastrous consequences of necrotizing myositis. Timely diagnosis, broad-spectrum antibiotic therapy, and aggressive surgical debridement of affected tissue are keys to the treatment of this serious, often life-threatening infection.

## Background

Streptococcal necrotizing myositis, also referred to as gangrenous myositis, is a very rare and severe soft tissue infection caused by group A β-hemolytic streptococcus [1, 2]. The incidence in Europe is estimated to be 0.22–0.6/100.000/year [3, 4]. Affected tissues predominately involve skeletal muscle and, eventually, superficial fascia and surrounding tissues. Fulminant, invasive muscle infections are, however, rare with no more than 30 cases reported over the last century [5]. The initial presentation is often non-specific until the rapidly progressing clinical course becomes apparent. A high mortality rate of 70–100% has been reported [6, 7].

## Case presentation

The presented case involves a 74-year-old male, Caucasian patient suffering from progressing swelling and gradually oncoming pain of the upper right extremity. No trauma was recollected. The symptoms had initially occurred the previous night. The patient denied any sore throat or congestion, dyspnea, chest pain or palpitations, abdominal pain, nausea, vomiting, diarrhea, dysuria, or rash.

The previous medical history included an excised soft tissue sarcoma of the left thigh with postoperative irradiation 5 years ago and regular follow-ups without pathological findings, as well as a benign prostatic hyperplasia and hyperuricemia. Regular medication included an uricostatic agent and an alpha receptor antagonist.

Physical examination indicated an edematous swelling of the right hand and forearm with superficial skin lesions of unknown cause, no reddening, a body temperature of 36.3 °C, a heart rate of 60 beats/min, an oxygen saturation of 94% with a respiratory rate of 19/min, and an arterial blood pressure of 156/69 mm/Hg with palpable pulses of the radial and ulnar artery, generalized tenderness upon palpation, and painfully restricted flection of all fingers. Laboratory tests showed a leukocytosis of 16,510/μl, and elevated levels for the C-reactive protein (CRP, 17.5 mg/dl) and creatine-kinase (CK, 669 U/l).

Computed tomographic imaging with application of a contrast agent (Ultravist^®^ 300, Bayer Vital, Leverkusen, Germany) showed a generalized subcutaneous edema as well as a subfascial fluid accumulation around the musculature of the fore- and upper arm, moreover a reduction of density along the brachialis muscle. The supplying arterial blood vessels (Aa. brachialis, radialis and ulnaris) presented regularly.

Within 6 h a severe deterioration of the clinical findings occurred with progressive swelling and development of a compartment syndrome of the arm: a palm sized livid discoloration located at the medial elbow occurred, multiple blisters, a hypoesthesia of all fingers and heavy pain resistant to analgesics treatment. The radial and ulnar artery were not palpable anymore and no arterial flow was detectable using doppler sonography.

With the clinical symptoms of a compartment syndrome of the upper extremity, the patient was consequently admitted to the theater for emergency surgical exploration, debridement as well as medial and lateral fasciotomy of the fore- and upper arm. An extensive excision of the necrotic tissue was performed. Blood cultures were taken as well as multiple tissue samples for histopathologic and microbiological analyses. Predominately, the forearm flexors appeared at risk (pronator teres, flexor carpi radialis and ulnaris, flexor digitorum superficialis and profundus, palmaris longus, pronator quadratus, flexor pollicis longus). The skin and underlying fascia were affected to a lower extend and excised to healthy tissue. The resulting soft tissue defect was temporarily covered with Epigard^®^.

After sample collection intravenous antibiotic therapy was started with Clindamycin and Penicillin G.

Postoperatively, the patient was transferred to the ICU. Microbiological analysis revealed group A β-hemolytic streptococcus, susceptible to Clindamycin and Penicillin G, histology a bacterial interstitial myositis with necrotic muscular fibers. Consecutively, the initially elevated blood infection parameters such as leukocyte count, and C-reactive protein levels were continuously decreasing.

However, the biochemical profile showed rising CK levels (5560 U/l) which were treated by fluid resuscitation and high-ceiling diuretics in the following to prevent an imminent crush syndrome. The morning after surgery CK levels had already decreased to 3537 U/l to further drop to 514 U/l the following day and 337 U/l another day later.

Three days after primary surgery, the patient was discharged from ICU and transferred to the general surgical ward for further observation and treatment.

**Fig. 1:**
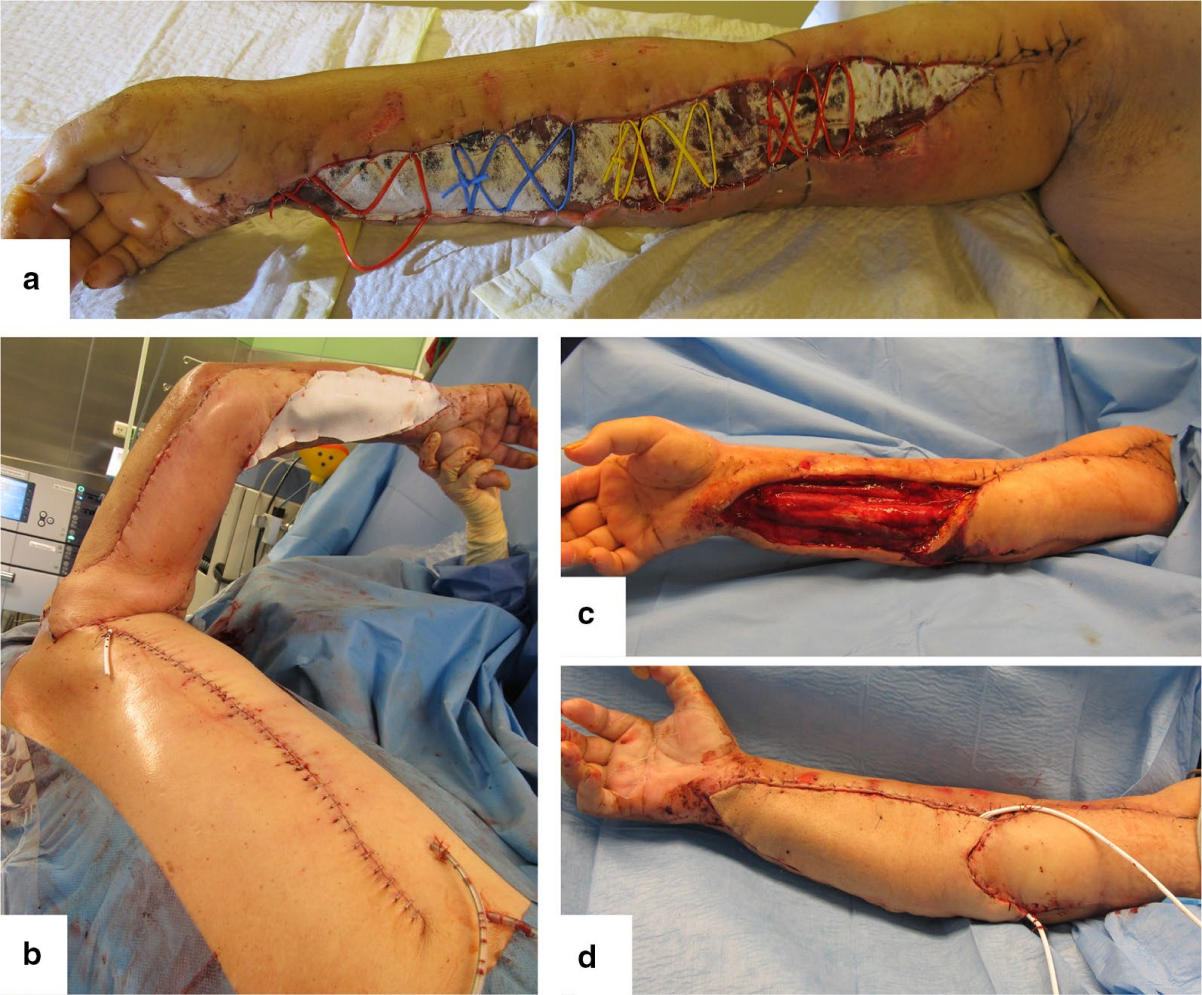
Soft tissue defect reconstruction applying dermatotraction (**a**), a pedicled, fasciocutaneous parascapular flap (**b**, **c**), and an ipsilateral anterolateral thigh flap (lateral circumflexing femoral artery perforator flap) (**d**)

The patient needed ten further visits to the theater for dressing changes but minimal further debridement prior to application of dermatotraction techniques to initiate wound closure as described previously [8]. Dermatotraction involved vessel loops anchored to alternate edges of the wound using skin staples. Remaining soft tissue defects of the upper arm were reconstructed with a pedicled, fasciocutaneous parascapular flap, the defects of the forearm with a free, ipsilateral anterolateral thigh flap (lateral circumflexing femoral artery perforator flap) (Fig. 1). On day 43 antibiotic therapy was consecutively switched to oral Ciprofloxacin and continued for 10 more days.

After a total of five sessions of plastic reconstructive surgery, the wounds healed uneventfully, and after physiotherapy the patient was discharged from hospital 63 days after admission. Follow-up appointments were scheduled on a monthly basis at first, with continuing physio- and ergotherapy.

**Fig. 2:**
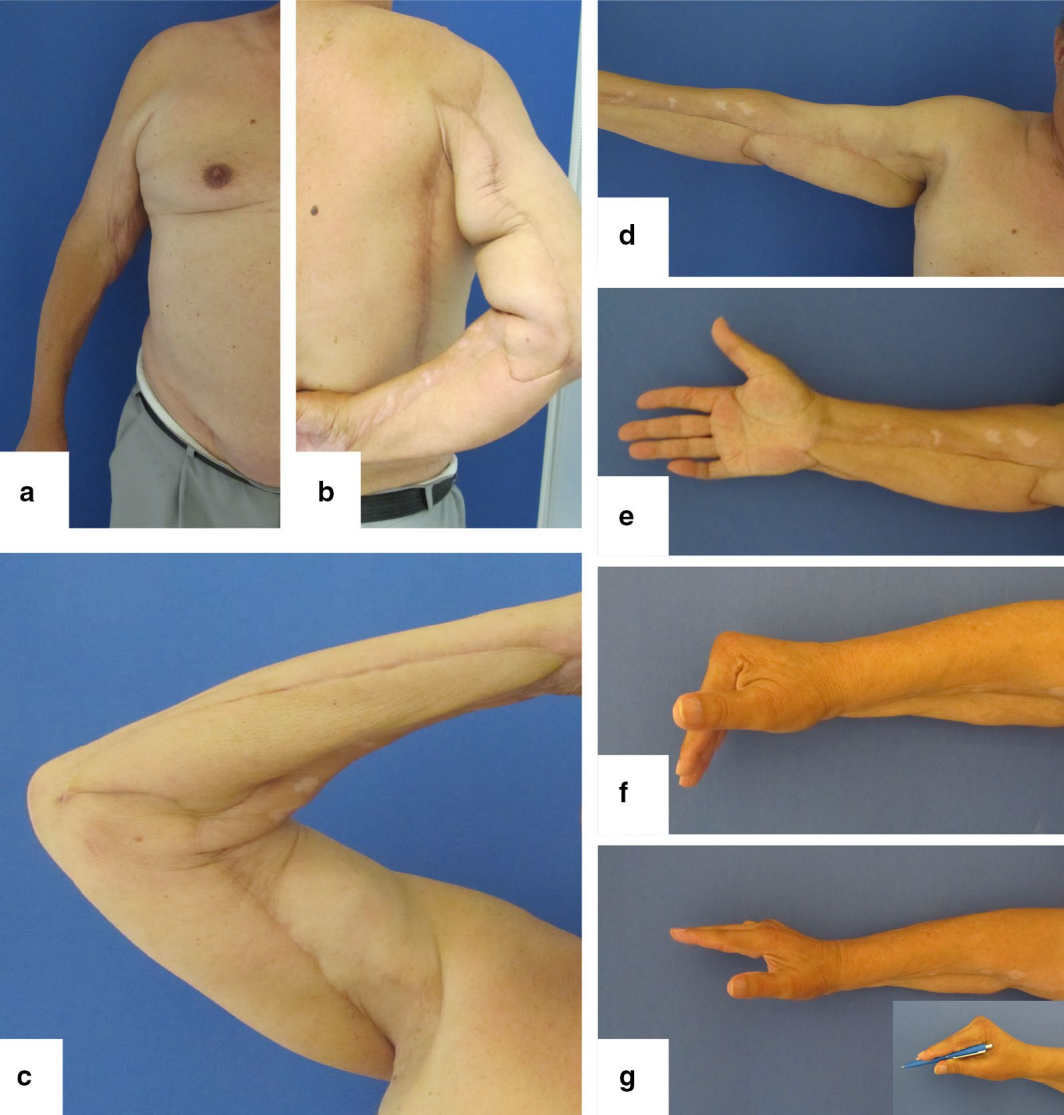
Functional outcome 14 months after discharge (**a**–**g**)

At the final follow-up 14 months after discharge, the flaps presented vital without any signs of inflammation or infection (Fig. 2). The range of motion of the affected joints was as follows: elbow: extension/flexion 0°/0°/95°, supination/pronation 60°/0°/60°. Wrist joint: extension/flexion 50°/0°/30°, abduction/adduction 20°/0°/10°. Metacarpophalangeal joints 2–4: extension/flexion 0°/20°/80°.

An active flexion of the proximal and distal interaphalangeal joints 2–4 was not possible, as the superficial and deep flexor muscles of the forearm were significantly weakened as a result of the extensive necrosectomy. The opposition of the thumb was also partially restricted, gripping and writing was however possible even though in a limited fashion.

## Discussion and conclusions

Group A β-hemolytic streptococcus (GAS) appears to be a highly virulent pathogen that is accounted responsible for a wide spectrum of clinical presentations. A wide variety of organs can be affected including the respiratory tract, skin, heart, and kidneys [9]. Rarely, GAS can cause highly invasive, rapidly progressive, life-threatening infections with extensive destruction of fascia or muscles in the form of necrotizing fasciitis (NF) and necrotizing myositis, respectively [2]. The increasing number of severe toxic streptococcal infections over the last 20 years has coincided with an increased prevalence of certain M protein serotypes, such as M1 and M3; and an increase in the number of GAS strains producing streptococcal pyrogenic exotoxin A and protease [10, 11].

Streptococcal necrotizing myositis (SNM) usually affects healthy, middle-aged patients and usually involves a single muscle group such as the thigh, calf, or arm muscle groups [12]. Normally, there is no history of penetrating trauma, and hematogenous spread from the pharynx is discussed as the most convincing way of transmission [7].

Necrotizing myositis is characterized by high rates of morbidity and mortality.

The rate of mortality is estimated to be 70–100% [6, 7]. This can be attributed to the often delayed diagnosis of a streptococcal myositis as the disease initially often causes unspecific symptoms similar to myalgia or a deep vein thrombosis [13, 14].

Generally, fulminant, invasive GAS muscle infections such as necrotizing fasciitis and streptococcal toxic shock syndrome are, however, rare with no more than 30 cases reported over the last century [5].

Besides an early correct diagnosis, high-dose intravenous antibiotic therapy, early surgical intervention with debridement or even amputation of the affected limb, and postoperative intensive care—especially in case of an imminent crush syndrome—represent the key aspects of SNM treatment [15].

In the present case, early diagnosis also proofed difficult. The clinical findings with a general swelling of the arm and elevated CRP and leukocyte levels in the peripheral blood primarily suggested a superficial soft tissue infection due to the obvious superficial skin lesions. The rapid progression of symptoms with disproportional pain and the development of a compartment syndrome in combination with the findings of CT imaging led to the diagnosis of a necrotizing soft tissue infection. As a result of early diagnosis (CT imaging) and local availability of the necessary resources (emergency unit, on site radiology and surgical stand-by duty, ICU) for sufficient patient treatment, a first surgical intervention could take place within 6 h after hospital admission.

Intraoperatively, primarily muscular tissue appeared affected with cyanotic livid necroses of the anterior (flexor) compartment of the forearm. Subcutaneous tissue and fascia as in case of necrotizing fasciitis were not involved, therefore missing the previously described lack of resistance of the normally adherent fascia to blunt dissection, distinctive, local edema (dishwater fluid) created by the subcutaneous tissue undergoing necrosis [16]. Microbiological analysis revealed the detection of group A β-hemolytic streptococcus which was in line with the histo-pathological findings of an interstitial myositis with fiber necrosis in presence of gram positive cocci. The clinical presentation, the initially elevated creatine-kinase levels, CT imaging, as well as the intraoperative findings together with the detection of GAS and a confirmed interstitial myositis consolidated the final diagnosis of a necrotizing myositis.

Closely following the above mentioned key aspects of SNM treatment greatly contributed to patient survival and preservation of the affected limb in the presented case. In addition, the outcome may also have been positively influenced by not administrating non-steroidal anti-inflammatory drug (NSAID) as recent scientific evidence suggests that nonselective NSAIDs accelerate disease progression and limit antibiotic efficacy in established GAS soft tissue infections [17].

In summary, the close interdisciplinary collaboration of orthopedic surgeons, microbiologists, pathologists, intensive care professionals, and plastic surgeons contributed to a successful treatment of this rare and dangerous disease.

Necrotizing myositis is a life-threatening soft-tissue infection unless diagnosed promptly and treated aggressively. Early diagnosis, aggressive surgical debridement, and broad-spectrum antibiotic therapy with a combination of penicillin and clindamycin is imperative. Early referral to theater for aggressive excision and debridement of infected tissues is crucial and early amputation must be considered if indicated.

## Abbreviations

NSAID: non-steroidal anti-inflammatory drug
GAS: group A β-hemolytic streptococcus
SNM: streptococcal necrotizing myositis
CT: computed tomography
CRP: C-reactive protein
NF: necrotizing fasciitis

## References

[1] Mearkle R, Saavedra-Campos M, Lamagni T, Usdin M, Coelho J, Chalker V (2017). Household transmission of invasive group A Streptococcus infections in England: a population-based study, 2009, 2011 to 2013. Euro Surveill.

[2] Rieger UM, Gugger CY, Farhadi J, Heider I, Andresen R, Pierer G (2007). Prognostic factors in necrotizing fasciitis and myositis: analysis of 16 consecutive cases at a single institution in Switzerland. Ann Plast Surg.

[3] Luca-Harari B, Darenberg J, Neal S, Siljander T, Strakova L, Tanna A (2009). Clinical and microbiological characteristics of severe Streptococcus pyogenes disease in Europe. J Clin Microbiol.

[4] Plainvert C, Doloy A, Loubinoux J, Lepoutre A, Collobert G, Touak G (2012). Invasive group A streptococcal infections in adults, France (2006–2010). Clin Microbiol Infect.

[5] Hasenboehler EA, McNair PJ, Rowland EB, Burch JM (2011). Necrotizing streptococcal myositis of an extremity: a rare case report. J Orthop Trauma.

[6] Weiss KA, Laverdiere M (1997). Group A Streptococcus invasive infections: a review. Can J Surg J.

[7] Adams EM, Gudmundsson S, Yocum DE, Haselby RC, Craig WA, Sundstrom WR (1985). Streptococcal myositis. Arch Intern Med.

[8] Harris I (1993). Gradual closure of fasciotomy wounds using a vessel loop shoelace. Injury.

[9] Wood TF, Potter MA, Jonasson O (1993). Streptococcal toxic shock-like syndrome. The importance of surgical intervention. Ann Surg.

[10] Salcido RS (2007). Necrotizing fasciitis: reviewing the causes and treatment strategies. Adv Skin Wound Care.

[11] Lacy MD, Horn K (2009). Nosocomial transmission of invasive group a streptococcus from patient to health care worker. Clin Infect Dis.

[12] Adair A, MacDonald L, Alasadi A, Holdsworth RJ (2009). Streptococcal myositis a surgical emergency. Surg.

[13] Kang N, Antonopoulos D, Khanna A (1998). A case of streptococcal myositis (misdiagnosed as hamstring injury). J Accid Emerg Med.

[14] Haywood CT, McGeer A, Low DE (1999). Clinical experience with 20 cases of group A streptococcus necrotizing fasciitis and myonecrosis: 1995 to 1997. Plast Reconstr Surg.

[15] Dalal M, Sterne G, Murray DS (2002). Streptococcal myositis: a lesson. Br J Plast Surg.

[16] Lemarechal A, Zundel S, Szavay P (2016). Pediatric necrotizing fasciitis: restitutio ad integrum after early diagnosis and aggressive surgical treatment. Eur J Pediatric Surg Rep.

[17] Bryant AE, Bayer CR, Aldape MJ, Stevens DL (2015). The roles of injury and nonsteroidal anti-inflammatory drugs in the development and outcomes of severe group A streptococcal soft tissue infections. Curr Opin Infect Dis.

